# Chronic cough hypersensitivity syndrome

**DOI:** 10.1186/1745-9974-9-14

**Published:** 2013-05-13

**Authors:** Alyn H Morice

**Affiliations:** 1 Cardiovascular & Respiratory Studies, Respiratory Medicine, Hull York Medical School, Castle Hill Hospital, Castle Road, Cottingham, East Yorkshire HU16 5JQ, UK

## Abstract

Chronic cough has been suggested to be due to three conditions, asthma, post nasal drip, and reflux disease. A different paradigm has evolved in which cough is viewed as the primary condition characterised by afferent neuronal hypersensitivity and different aspects of this syndrome are manifest in the different phenotypes of cough. There are several advantages to viewing cough hypersensitivity as the unifying diagnosis; Communication with patients is aided, aetiology is not restricted and therapeutic avenues opened. Cough Hypersensitivity Syndrome is a more applicable label to embrace the clinical manifestations of this disabling disease.

## Introduction

Our continuing difficulty in dealing with patients presenting with chronic cough is an example of an established dogma failing to measure up to awkward clinical facts. In the 1980s patients presenting with isolated chronic cough were suggested to fall into one of three diagnostic categories. Indeed, this hypothesis was crystallised into ‘the diagnostic triad of cough’ [1]. Cough was either due to a form of asthma, gastroesophageal reflux disease, or a nebulous and poorly defined condition variously called, Post Nasal Drip Syndrome or the Upper Airways Cough Syndrome [2]. Unfortunately, for those promulgating this paradigm patients presenting to the clinic often failed to fit into these diagnostic categories. The term idiopathic cough was duly coined to overcome the difficulties arising from the failure to pigeonhole such awkward patients [3].

To many working in the field this classification was deeply unsatisfactory. Firstly, when one of the three diagnostic labels was attached to a patient further investigation frequently revealed a grossly atypical pattern of disease. Thus, patients with ‘asthmatic cough’ sometimes did not wheeze, a symptom most would regard as sine qua non of asthma. These patients with cough variant or cough predominant asthma were suggested to have a differential location of the inflammation within the airway. However, an even more bizarre form of ‘asthma’, known as eosinophilic bronchitis, was also clearly a significant cause of cough in the clinic [4]. Here there is no bronchial hyperresponsiveness but evidence, usually obtained at induced sputum, of eosinophilic inflammation within the airways. Is this a form of asthma? Some would suggest that this is a separate condition. In the clinic however patients frequently straddle diagnostic boundaries and the further description of subtypes expands the numbers of “diseases” causing cough.

Secondly, there are patients whose predominant symptom is cough but clearly have conditions which are a recognised illness, such as pulmonary fibrosis due to interstitial lung disease or non cystic fibrosis bronchiectasis. In some patients their chronic cough, on detailed history, is virtually identical in nature and in associated features to the cough seen in patients with other forms of chronic cough [5]. Does the illness cause the cough or is the cough (through its underlying aetiology) actually the cause of the illness?

In an attempt to clarify this state of affairs the concept arose that the similarities in the clinical features of patients presenting with a chronic cough outweighed the differences. Thus, cough became to be viewed in a completely different paradigm. In the majority of patients with chronic cough it was suggested that there were not a series of individual diseases leading to the symptom but it was rather that there was a single underlying condition, chronic cough, which gave rise to a variety of different phenotypes. Since virtually all patients exhibit a hypersensitivity of the cough reflex the term Cough Hypersensitivity Syndrome was coined as an overarching diagnostic label [6,7]. As with any attempt to characterise and codify the clinical world the Cough Hypersensitivity Syndrome does have a number of drawbacks [8]. However, the greater understanding of the diagnosis and symptom profile exhibited by the patient, coupled with insights into the epidemiology, management and potential future developments have established Cough Hypersensitivity as the most accurate and convenient diagnostic grouping for patients suffering with chronic cough. In the clinic adopting the approach of establishing the Cough Hypersensitivity Syndrome as the primary diagnosis and then recognising the different phenotypes of allergic upper and lower airway inflammation and in the majority non allergic inflammatory change provides lucidity in both management and therapy.

## Evidence for cough hypersensitivity

That hypersensitivity of the cough reflex occurs during an upper respiratory tract infection is a universal experience. During a cough/cold we all experience bouts of coughing as the result of minor environment insults, such as change in temperature or exposure to noxious stimuli like cigarette smoke. Objective evidence of this hypersensitivity relies on challenge experiments. A shift in the cough dose response curve with a lower threshold in URTI, has been demonstrated to capsaicin challenge [9,10]. Recovery of the cough reflex to a more normal level is seen as the infection abates. I suggest that cough reflex hypersensitivity induced by virus is a fundamental part of the pathogenesis of URTI enabling the viruses to disseminate themselves through the population using droplet transmission. A similar mechanism for manual transmission exists in the coryza so characteristic of human cough/cold. Experimental studies done by Tyrrel in the Common Cold Research Unit in the 1950s clearly demonstrate this as the major mechanism of viral transmission [11].

**Figure 1:**
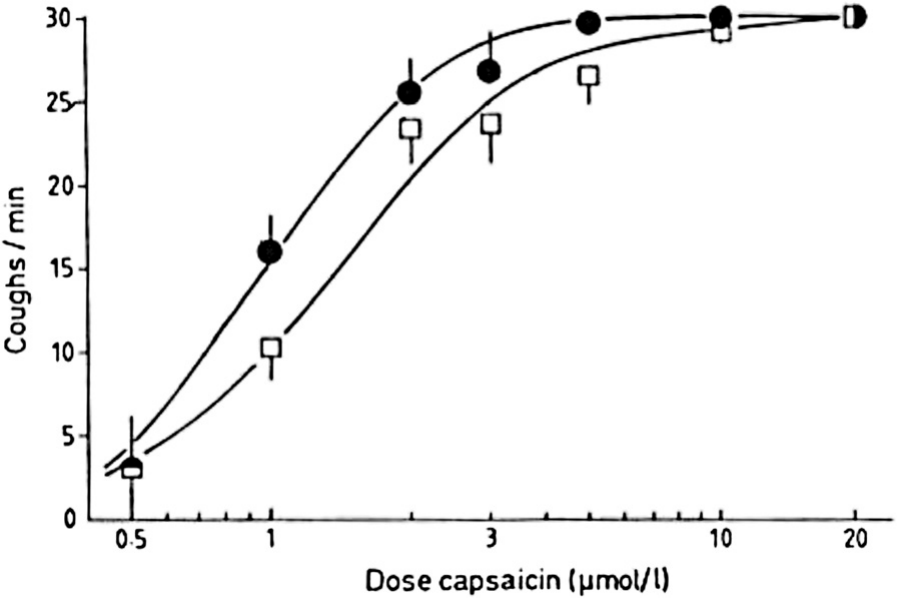
**Capsaicin dose response curve on captopril (squares) or placebo (circles).** Leftward shift indicates an increased cough sensitivity on ACE inhibitor.

Whether these observations of afferent neuronal hypersensitivity are relevant to subjects with a chronic cough have been difficult to prove with certainty and still cause manifest confusion in the minds of clinicians and in the published literature. The reason for this lack of clarity is that unlike the bronchial hyperresponsiveness seen in the asthmatic population where increased bronchomotor tone is reflected in bronchoconstriction to, for example methacholine, cough reflex hypersensitivity as manifest by increased response to capsaicin or acid stimulus usually still lies within the wide normal range. Thus, bronchial hyperresponsiveness to methacholine is demonstrated by shift in pc20 from a typical normal value of >16 mg/ml to that below 4 mg/ml. In capsaicin responsiveness and individual subject may show a C5 response of 1 micromolar capsaicin, which for them corresponds to hyperresponsiveness but this value is well within the normal range seen in the general population. This individual’s normal value may be 30 micromolar and this is only revealed by the successful treatment of their chronic cough. Cough hypersensitivity is therefore unreliable as a test of abnormality for an individual patient. Cough hypersensitivity can be revealed in populations as illustrated in Figure 1[12]. A shift in the capsaicin sensitivity induced by ACE inhibitors is seen but the overwhelming majority of individuals remain within the broad population normal range. Thus, in the clinic there is very limited utility in performing cough challenge to demonstrate cough hypersensitivity since it is of no value in diagnosis and is only roughly correlated with the clinical perception of cough.

## Epidemiological evidence

**Figure 2:**
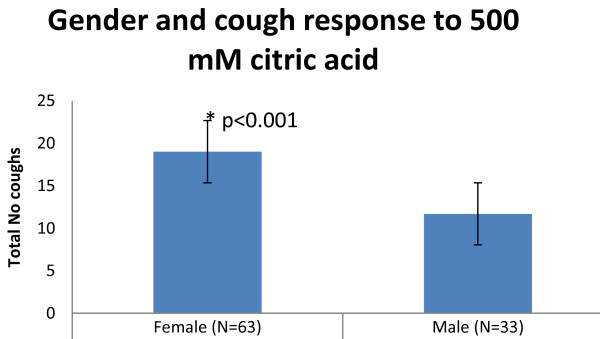
Mean cough response to citric acid in men vs women.

The association of cough hypersensitivity with clinically important chronic cough is perhaps best illustrated by the epidemiological evidence of cough hypersensitivity in the sexes. Several studies show that women have heightened cough reflex to inhalation challenge with capsaicin, citric acid, and tartaric acid [13,14]. However this finding is not universal. For example we have recently analysed a population of normal volunteers 39 males mean age 31 and 63 females mean age 37 and found a near doubling of cough evoked by inhalation of 500 mM citric acid (Figure 2). Whereas in a similar volunteer pool of 54 females, mean age 34 and 46 males, mean age 31 there was no significant difference found in capsaicin dose response with C2 (11 and 15 uM) and C5 (39 and 66 uM) values respectively being similar between the sexes. Given the previously described wide range in cough sensitivity then large numbers are required to detect any significant population differences with certainty.

This female hypersensitivity appears to occur after puberty, since girls and boys have the same reflex sensitivity [15,16]. It has been suggested that cough hypersensitivity in women is an evolutionary mechanism to protect against aspiration during pregnancy. Preliminary evidence from functional magnetic imaging studies suggests that cough centres are enlarged in women. This sex difference in cough reflex sensitivity is reflected in the population attending cough clinics. In a demographic survey of over 5000 presentations with chronic cough to specialist services women attended twice as frequently as men, with a peak age of presentation in the 5^th^ and 6^th^ decade. Here women also have a heightened cough reflex sensitivity to capsaicin compared to men [17].

## Mechanism of cough hypersensitivity

The mechanism whereby the hypersensitivity state within the upper airways is produced is currently unknown. Several possible mechanisms have been hypothesised. A central response may well underlie the sex related difference to cause of the female preponderance. However, it is unlikely to explain the local effects seen in airway disease. Thus, patients with eosinophilic bronchitis appears to have a particular form of mast cell infiltration associated with airway nerves [18]. In contrast, in classic asthma the same infiltrate is distributed within the airway smooth muscle. Thus, targeting of inflammation to particular airway structures may be responsible for some of the different phenotypes seen within the spectrum of asthmatic cough. The distribution of nerves may well be altered by disease. In a careful biopsy study Groneberg et al [19] showed that there was an increase in TRPV1 containing subepithelial sensory nerves within the bronchial wall of chronic cough patients. However, Mitchell et al [20] did not find this. Sampling error is bound to be an important factor in studies of small numbers in what is a highly variable autonomic system in human lung. That proinflammatory mediators can induce TRP receptor function has been demonstrated in primary human lung cell culture [21].

Within the vagus nerve various mechanisms of hypersensitivity have been established, including that induced by prostaglandins, particular PGE2 [22]. In the nodose and jugular ganglia work from John’s Hopkins has demonstrated that a distinct subset of neurones mediate the cough response [23] and these may be responsible of some manifestations of cough hypersensitivity.

## Pathogenesis of cough hypersensitivity

It is clear that a wide range of different insults may lead to the inflammation and epithelial damage required to produce afferent sensory hypersensitivity of the upper airways. Simple thermal or toxic damage induced by, for example smoke inhalation or exposure to extreme cold dry air is responsible for a number of clinical scenarios. Human models of this are produced in the industrial setting. Thus, exposure to hot acidic gas in bottle manufacturing leads to an increase in cough reflex hypersensitivity and an increase of cough related symptoms [24]. Locally the factory was known as the ‘asthma factory’, although investigation revealed cough hypersensitivity rather than bronchial hyperresponsiveness.

A major and much overlooked cause of cough hypersensitivity is gaseous non acid reflux. Because of the lack of associated classic reflux symptoms of heartburn and regurgitation this form of reflux, although described over 150 years ago as causing typical upper respiratory symptoms [25] has been largely missed. A questionnaire (HARQ) has been developed to determine the associated features of reflux induced cough [26]. Because of the lack of classic symptoms the syndrome has been entitled ‘silent reflux’ by the ENT surgeons since there is associated loss of voice [27]. However, it is perhaps not an appropriate term for a syndrome causing cough. Because of the lack of acid, conventional oesophageal studies, such as 24 hour pH monitoring are uninformative. The single most useful investigation is high resolution manometry, which provides information as to the underlying neuromechanical defect of oesophageal function leading to excessive gaseous reflux [28].

## Cough hypersensitivity as a disease

The paradigm shift from regarding cough as a symptom of various diseases into cough as a disease with different facets allows for a number of advantages in both diagnosis and therapy of this condition.

Firstly, the idea of cough hypersensitivity as the disease removes the necessity to have a subgroup of chronic coughers who have idiopathic cough. The patient does have a disease; it is just that its origin may be mysterious. If a considerable portion of patients with cough hypersensitivity have occult airway reflux then the degree of certainty for individual clinicians is dependent on their acceptance of the strength of evidence supporting the diagnosis. Thus, the author would place many patients in the category of cough hypersensitivity syndrome secondary to airway reflux whereas others, who have less agreement with this as an aetiological mechanism, would place more patients in cough hypersensitivity syndrome of unknown aetiology. Cough hypersensitivity syndrome is thus a unifying concept allowing differing opinions to be held within differing degrees of precision as to the pathobiological basis of the illness.

The second great strength of cough hypersensitivity as a diagnosis is that by concentrating on a unifying pathological feature it points the way to possible therapeutic avenues which would not otherwise be revealed.

Thirdly, communicating with the patient is facilitated by the diagnosis of the cough hypersensitivity syndrome. Many patients in the clinic express their extreme frustration in the lack of understanding and the lack of a firm diagnosis provided by the profession [29]. By giving the syndrome a name and understanding the distinct epidemiological features outlined above allows the physician to communicate our understanding of what is known about the condition. Too often an isolated chronic cough is dismissed as psychogenic or worse, when objective cough counting clearly demonstrates the extreme frequency of coughing paroxysms and explains the great distress suffered by the patient [30].

## Competing interests

The authors declares that he has no competing interests.

## References

[B1] Palombini BC Villanova CA Araujo E Gastal OL Alt DC Stolz DP Palombini CO A pathogenic triad in chronic cough: asthma, postnasal drip syndrome, and gastroesophageal reflux disease Chest 1999 116 279 284 10.1378/chest.116.2.279 10453852

[B2] Pratter MR Chronic upper airway cough syndrome secondary to rhinosinus diseases (previously referred to as postnasal drip syndrome): ACCP evidence-based clinical practice guidelines Chest 2006 129 63S 71S 10.1378/chest.129.1_suppl.63S 16428694

[B3] McGarvey LP Idiopathic chronic cough: a real disease or a failure of diagnosis? Cough 2005 1 9 10.1186/1745-9974-1-9 16270939 PMC1277011

[B4] Brightling CE Ward R Goh KL Wardlaw AJ Pavord ID Eosinophilic bronchitis is an important cause of chronic cough Am J Respir Crit Care Med 1999 160 406 410 10.1164/ajrccm.160.2.9810100 10430705

[B5] Fahim A Dettmar PW Morice AH Hart SP Gastroesophageal reflux and idiopathic pulmonary fibrosis: a prospective study Medicina (Kaunas) 2011 47 200 205 21829051

[B6] Chung KF Chronic 'cough hypersensitivity syndrome': a more precise label for chronic cough Pulm Pharmacol Ther 2011 24 267 271 10.1016/j.pupt.2011.01.012 21292019

[B7] Morice AH The cough hypersensitivity syndrome: a novel paradigm for understanding cough Lung 2010 188 S87 S90 19809853 10.1007/s00408-009-9185-z

[B8] Morice AH McGarvey LP Dicpinigaitis PV Cough hypersensitivity syndrome is an important clinical concept: a pro/con debate Lung 2012 190 3 9 10.1007/s00408-011-9351-y 22186805

[B9] Dicpinigaitis PV Bhat R Rhoton WA Tibb AS Negassa A Effect of viral upper respiratory tract infection on the urge-to-cough sensation Respir Med 2011 105 615 618 10.1016/j.rmed.2010.12.002 21185164

[B10] O’ Connell F Thomas VE Studham JM Pride NB Fuller RW Capsaicin cough sensitivity increases during upper respiratory infection Resp Med 1996 90 279 286 10.1016/S0954-6111(96)90099-2 9499812

[B11] Tyrrell DA A view from the common cold unit Antiviral Res 1992 18 105 125 10.1016/0166-3542(92)90032-Z 1329647 PMC7133934

[B12] Morice AH Lowry R Brown MJ Higenbottam T Angiotensin converting enzyme and the cough reflex Lancet 1987 2 1116 1118 2890021 10.1016/s0140-6736(87)91547-9

[B13] Fujimura M Sakamoto S Kamio Y Matsuda T Sex difference in the inhaled tartaric acid cough threshold in non-atopic healthy subjects Thorax 1990 45 633 634 10.1136/thx.45.8.633 2402729 PMC462649

[B14] Fujimura M Kasahara K Kamio Y Naruse M Hashimoto T Matsuda T Female gender as a determinant of cough threshold to inhaled capsaicin Eur Respir J 1996 9 1624 1626 10.1183/09031936.96.09081624 8866583

[B15] Chang AB Phelan PD Roberts RGD Robertson CF Capsaicin cough receptor sensitivity test in children Eur Respir J 1996 9 2220 2223 10.1183/09031936.96.09112220 8947063

[B16] Varechova S Plevkova J Hanacek J Tatar M Role of gender and pubertal stage on cough sensitivity in childhood and adolescence J Physiol Pharmacol 2008 59 Suppl 6 719 726 19218699

[B17] Kastelik JA Thompson RH Aziz I Ojoo JC Redington AE Morice AH Sex-related differences in cough reflex sensitivity in patients with chronic cough Am J Respir Crit Care Med 2002 166 961 964 10.1164/rccm.2109061 12359654

[B18] Brightling CE Bradding P Symon FA Holgate ST Wardlaw AJ Pavord ID Mast cell infiltration of airway smooth muscle in asthma N Engl J Med 2002 346 1699 1705 10.1056/NEJMoa012705 12037149

[B19] Groneberg DA Niimi A Dinh QT Cosio B Hew M Fischer A Chung KF Increased expression of transient receptor potential vanilloid-1 in airway nerves of chronic cough Am J Respir Crit Care Med 2004 170 1276 1280 10.1164/rccm.200402-174OC 15447941

[B20] Mitchell JE Campbell AP New NE Sadofsky LR Kastelik JA Mulrennan SA Compton SJ Morice AH Expression and characterization of the intracellular vanilloid receptor (TRPV1) in bronchi from patients with chronic cough Exp Lung Res 2005 31 295 306 10.1080/01902140590918803 15962710

[B21] Sadofsky LR Ramachandran R Crow C Cowen M Compton SJ Morice AH Inflammatory stimuli up-regulate transient receptor potential vanilloid-1 expression in human bronchial fibroblasts Exp Lung Res 2012 38 2 75 81 10.3109/01902148.2011.644027 22242698

[B22] Belvisi MG Dubuis E Birrell MA Transient receptor potential A1 channels: insights into cough and airway inflammatory disease Chest 2011 140 1040 1047 10.1378/chest.10-3327 21972382 PMC3186687

[B23] Canning BJ Mazzone SB Meeker SN Mori N Reynolds SM Undem BJ Identification of the tracheal and laryngeal afferent neurones mediating cough in anaesthetized guinea-pigs J Physiol 2004 557 543 558 10.1113/jphysiol.2003.057885 15004208 PMC1665106

[B24] Gordon SB Curran AD Turley A Wong CH Rahman SN Wiley K Morice AH Glass bottle workers exposed to low-dose irritant fumes cough but do not wheeze Am J Respir Crit Care Med 1997 156 206 210 10.1164/ajrccm.156.1.9610042 9230749

[B25] Congreve GT On consumption of the lungs, or, Decline and its successful treatment: showing that formidable disease to be curable in all its stages: with observations on coughs, colds, asthma, chronic bronchitis etc 1881 London: Author and Eliot Stock

[B26] Morice AH Faruqi S Wright CE Thompson R Bland JM Cough hypersensitivity syndrome: a distinct clinical entity Lung 2011 189 73 79 10.1007/s00408-010-9272-1 21240613

[B27] Belafsky PC Postma GN Koufman JA Validity and reliability of the reflux symptom index (RSI) J Voice 2002 16 274 277 10.1016/S0892-1997(02)00097-8 12150380

[B28] Vardar R Sweis R Anggiansah A Wong T Fox MR Upper esophageal sphincter and esophageal motility in patients with chronic cough and reflux: assessment by high-resolution manometry Dis Esophagus 2012 26 3 219 225 22591118 10.1111/j.1442-2050.2012.01354.x

[B29] Everett CF Kastelik JA Thompson RH Morice AH Chronic persistent cough in the community: A questionnaire survey Cough 2007 3 5 10.1186/1745-9974-3-5 17381836 PMC1847685

[B30] Decalmer SC Webster D Kelsall AA McGuinness K Woodcock AA Smith JA Chronic cough: how do cough reflex sensitivity and subjective assessments correlate with objective cough counts during ambulatory monitoring? Thorax 2007 62 329 334 10.1136/thx.2006.067413 17101736 PMC2092471

